# Fever case management at private health facilities and private pharmacies on the Kenyan coast: analysis of data from two rounds of client exit interviews and mystery client visits

**DOI:** 10.1186/s12936-018-2267-8

**Published:** 2018-03-13

**Authors:** Stephen Poyer, Anne Musuva, Nancy Njoki, Robi Okara, Andrea Cutherell, Dana Sievers, Cristina Lussiana, Dorothy Memusi, Rebecca Kiptui, Waqo Ejersa, Stephanie Dolan, Nicole Charman

**Affiliations:** 1 Population Services International, Nairobi, Kenya; 2 Population Services Kenya, Nairobi, Kenya; 3 Population Services International, Washington, DC, USA; 4 National Malaria Control Programme, Ministry of Health, Nairobi, Kenya

**Keywords:** Malaria, Case management, Diagnosis, Rapid diagnostic test, Private sector, Registered pharmacy

## Abstract

**Background:**

Private sector availability and use of malaria rapid diagnostic tests (RDTs) lags behind the public sector in Kenya. Increasing channels through which quality malaria diagnostic services are available can improve access to testing and help meet the target of universal diagnostic testing. Registered pharmacies are currently not permitted to perform blood tests, and evidence of whether malaria RDTs can be used by non-laboratory private providers in line with the national malaria control guidelines is required to inform ongoing policy discussions in Kenya.

**Methods:**

Two rounds of descriptive cross-sectional exit interviews and mystery client surveys were conducted at private health facilities and registered pharmacies in 2014 and 2015, 6 and 18 months into a multi-country project to prime the private sector market for the introduction of RDTs. Data were collected on reported RDT use, medicines received and prescribed, and case management of malaria test-negative mystery clients. Analysis compared outcomes at facilities and pharmacies independently for the two survey rounds.

**Results:**

Across two rounds, 534 and 633 clients (including patients) from 130 and 120 outlets were interviewed, and 214 and 250 mystery client visits were completed. Reported testing by any malaria diagnostic test was higher in private health facilities than registered pharmacies in both rounds (2014: 85.6% vs. 60.8%, p < 0.001; 2015: 85.3% vs. 56.3%, p < 0.001). In registered pharmacies, testing by RDT was 52.1% in 2014 and 56.3% in 2015. At least 75% of test-positive patients received artemisinin-based combination therapy (ACT) in both rounds, with no significant difference between outlet types in either round. Provision of any anti-malarial for test-negative patients ranged from 0 to 13.9% across outlet types and rounds. In 2015, mystery clients received the correct (negative) diagnosis and did not receive an anti-malarial in 75.5% of visits to private health facilities and in 78.4% of visits to registered pharmacies.

**Conclusions:**

Non-laboratory staff working in registered pharmacies in Kenya can follow national guidelines for diagnosis with RDTs when provided with the same level of training and supervision as private health facility staff. Performance and compliance to treatment recommendations are comparable to diagnostic testing outcomes recorded in private health facilities.

## Background

Since 2010 the World Health Organization (WHO) has recommended that every suspected malaria case be confirmed by parasitological testing using a quality rapid diagnostic test (RDT) or by microscopy, and that uncomplicated *Plasmodium falciparum* malaria be treated with artemisinin-based combination therapy (ACT) [1]. The use of high-quality diagnostic testing for malaria can improve the targeting of anti-malarials and reduce wastage, lead to the correct assessment and treatment of non-malaria febrile illnesses, contribute to more accurate case detection and reporting, and reduce the selection pressure for anti-malarial drug resistance [2, 3].

Universal confirmatory diagnosis was introduced in Kenya through the 2009–2017 National Malaria Strategy, accompanied by a large-scale roll-out of RDTs to public facilities in 2012 [4]. By 2013, large gains had been recorded in public sector readiness to test and treat: availability of RDTs at public health facilities had increased from less than 10% in 2010 to 70, and 58% of patients presenting with fever in 2013 were being tested for malaria [5]. Among tested cases in 2013, 50% were treated according to the test result, up from 16% in 2010 [5]. Similarly, large improvements have been seen in reducing stock-outs of ACT, and in indicators related to the provision of training and supervision [5]. Following a mid-term review, the National Malaria Strategy was updated in 2014 to include a commitment to increasing engagement and coordination with private health providers, and ensuring access to and use of affordable diagnostic tests in the private sector [4]. The private sector covers a diverse range of providers in Kenya, including private for-profit and not-for-profit health facilities, registered and unregistered pharmacies, and general retail shops. Little is known about the quality of fever case management in the sector that is the source of care for 25% of fever cases in children under 5 years of age [6], and accounts for over 60% of anti-malarials sold or distributed in Kenya [7]. Availability of any diagnostic test (microscopy or RDT) was 45% in private health facilities and 18% in registered pharmacies in December 2011, though RDT availability was lower (7 and 7% respectively) [7]. In comparison, ACT availability was high in both channels: among outlets with anti-malarials in stock, 84% of private health facilities and 95% of registered pharmacies stocked any ACT [7].

Achieving universal access to malaria diagnosis and treatment requires both closing the testing availability gap between public and private sectors, and ensuring high-quality fever case management is available from all providers with the mandate to test and treat. Increasing the range of providers that can offer quality malaria diagnostic services is one way to improve access to testing and help meet the target of universal testing of fever for malaria. Private health facilities in Kenya are permitted to test for malaria by microscopy and RDT under regulations implemented by the Pharmacy and Poisons Board (PPB) and the Kenya Medical Laboratory Technicians and Technologists Board (KMLTTB). However, registered pharmacies have not historically been allowed to perform blood tests, as they do not typically employ registered laboratory technologists who are permitted to do so. Evidence is thus required to show whether malaria RDTs can be conducted safely and to a given standard by the non-laboratory cadre of providers common in these pharmacies.

Between 2013 and 2016 Population Services Kenya (PS Kenya) worked with the National Malaria Control Programme (NMCP) of the Kenyan Ministry of Health (MOH) on a Unitaid-funded partnership to stimulate the creation of a private sector market for quality-assured RDTs. Additional funding was provided by the UK Department for International Development (DFID) to expand the project’s geographic scope. The project aimed to improve access to quality-assured RDTs through increasing availability, increasing demand for diagnostic testing, and improving the quality of private sector fever case management. Implementation took place on the Kenyan coast at private health facilities and registered pharmacies (under a waiver from the KMLTTB). This paper describes the main project activities undertaken, and presents key fever case management findings from analysis of client exit interview and mystery client data collected at private health facilities and registered pharmacies in 2014 and 2015, 6 and 18 months after project implementation began.

## Methods

### Study setting

The studies were undertaken in Kilifi, Mombasa and Kwale counties in Kenya, in the coastal endemic zone. Malaria transmission is stable throughout the year and prevalence of *P. falciparum* was 8% among children aged 6 months to 14 years in 2015 [6]. Kwale and Kilifi counties are predominantly rural while Mombasa County is urban. The coastal area has a humid tropical climate with high temperatures and rainfall throughout the year. There are two main rainy seasons: the long rains that occur from April to June and the short rains that occur from October to December. In 2014, health service structures for Kilifi, Mombasa and Kwale counties respectively included: 47, 41 and 50 public health facilities; 182, 194 and 48 private health facilities; and 29, 241 and 17 registered pharmacies.

### Main programme activities during 2013–2016

Outlet mapping was conducted in August 2013 and provided the PS Kenya implementation team with a census of all private health facilities and pharmacies in the project area. In total, 682 active sites were located, and 524 outlets (218 private health facilities and 306 pharmacies) agreed to respond to a short questionnaire on business practises to determine eligibility. With input from the MOH, outlets were eligible for inclusion in the project if they (i) had a current valid registration certificate; (ii) belonged to a cadre already permitted to perform malaria RDTs or for which the project would request special permission; (iii) expressed a willingness to stock and perform RDTs, and; (iv) agreed to routinely submit case monitoring data to the implementation team. Among interviewed outlets, 77% of private health facilities (N = 218) and 75% of pharmacies (N = 306) had a current valid registration certificate. The majority of private health facilities (86%) reported offering diagnostic testing for malaria and 36% had RDTs available at the time of the survey, while 15% of interviewed pharmacies reported offering diagnostic testing and 12% had RDTs in stock for sale. In total, 317 outlets (142 private health facilities and 175 registered pharmacies) met all eligibility criteria and agreed to enrol in the project. Among eligible outlets, project enrolment took two approaches: in Kwale County, outlets were enrolled in one batch in late 2013; in Kilifi and Mombasa counties, outlet enrolment was a continuous process through the end of 2014. Staff at enrolled private health facilities were typically nurses and medical officers, while providers at pharmacies were typically pharmaceutical technologists and pharmacists. In June 2014, the KLMTTB approved NMCP’s request to allow PS Kenya to introduce RDTs at registered pharmacies within the project area. Unpublished results from a household survey on fever-case management conducted in project areas in December 2013 (before project implementation) suggest no more than 4% of febrile patients of all ages received an RDT when visiting a private sector source for advice or treatment.

The majority of provider training took place in March 2014. By June 2014, providers from 241 enrolled outlets had been trained on RDT use and fever case management by a team comprising county health officials and staff from the NMCP and PS Kenya. Providers from outlets in Kilifi and Mombasa enrolling after June 2014 were trained by the end of 2014. Training materials were adapted from the existing public-sector curriculum by NMCP staff with support from project partners PS Kenya, WHO, and Johns Hopkins Bloomberg School of Public Health (JHSPH). Materials and Standard Operational Procedures (SOPs) covered malaria epidemiology in Kenya, correct RDT procedure, case management of test-positive cases with ACT, and management of test-negative cases (defined for private health facilities as further investigations and for registered pharmacies as referral to a health facility). Separate trainings were held for providers from private health facilities and registered pharmacies, but both groups followed the same curriculum and both sessions lasted 3 days.

PS Kenya conducted a broad market analysis of private sector diagnostic testing in March 2014 which informed the initial RDT quantification calculations and pricing strategy. Prior to purchasing RDTs to prime the market, the country team reviewed the National RDT Specifications with support from NMCP and project partners WHO and the Foundation for Innovative New Diagnostics (FIND), and ensured tender specifications matched WHO recommendations for procurement criteria. The RDTs procured for the project were *CareStart Malaria HRP2 (Pf)* (catalogue number G0141), manufactured by AccessBio. Pre- and post-shipment testing for all procured lots was carried out at the Research Institute for Tropical Medicine (RITM) in Manila, Philippines and the Institute Pasteur of Cambodia (IPC) in Phnom Penh, and managed by FIND. Procured RDTs were promoted by local medical detailers and sold directly to project outlets at a median price per kit of USD 0.39 equivalent for a hospital pack of 25 RDTs with a shared buffer vial and USD 0.64 for a single kit packaged with an individual buffer vial. The suggested retail price was equivalent to USD 0.80 for an RDT from a hospital pack and USD 1.00 for a single pack RDT. Participating outlets were supplied with gloves and sharps boxes free-of-charge for the duration of the project by the project, and registered pharmacies liaised with local health facilities that provided biomedical waste disposal. With one exception, the project did not generally intervene in the supply of anti-malarials available to or stocked by providers. In mid-2015 30,000 ACT doses were procured for direct sale to project outlets to respond to ACT stock outs occurring between Global Fund rounds.

All providers received routine supportive supervision visits throughout the life of the project, with supervisors observing provider–client interactions, assessing providers’ RDT performance and providing immediate feedback. PS Kenya developed and conducted behaviour change communication (BCC) activities based on local market research to increase client demand for RDTs. Messaging highlighted that “fever is not equal to malaria, confirm with an RDT” and messages were delivered through radio, printed materials, and interpersonal and small group communication sessions.

### Study design

The studies utilized repeat cross-sectional client exit interview and mystery client visits at private health facilities and registered pharmacies participating in the project (without controls). For donor reporting requirements the studies had two geographic domains: Kilifi and Mombasa counties combined, and Kwale County. Studies were powered to estimate aggregate private sector performance (private health facilities and registered pharmacies combined) in each domain in each round. Specific study design, sample size, sampling and measurement details are provided separately for exit interviews and mystery client visits below.

### Client exit interviews

#### Study design, sample size and sampling

Repeat cross-sectional cluster surveys of adult clients seeking treatment for themselves, or on behalf of someone else, at participating private health facilities and registered pharmacies were conducted in September–October 2014 (6 months following provider training) and October–November 2015 (18 months following provider training). The sampling frames for each round comprised all registered private health facilities and registered pharmacies actively participating in the project as of September 2014 (167 outlets) and June 2015 (146 outlets). Active outlets were those that had placed at least one order for RDTs through the project following provider training and had not dropped out of the project. The study’s objective was to estimate the level of use of diagnostic tests generally, and RDTs specifically, by providers at active outlets at each round. Client sample sizes were calculated by geographic domain to provide estimates of the project indicator *proportion of patients seeking treatment for fever that received an RDT*, with a confidence level of 95%, an estimated design effect of 1.5 due to clustering at the outlet-level, and a margin of error of 7 percentage points in Kwale and 5 percentage points in Kilifi/Mombasa. The project target of 30% was used as the hypothesized testing level for both survey rounds. These calculations led to required samples of 247 fever clients in Kwale and 484 fever clients in Kilifi/Mombasa. This sample size was operationalized using estimates of the proportion of clients presenting with a history of fever, the number of patients visiting in-person and assuming a 10% refusal rate. The number of outlets selected as study sites for each round followed a pragmatic approach driven by (i) the number of participating outlets in each domain, (ii) routine monitoring data on client loads, and (iii) a desire to minimize the number of data collection days at any one outlet. A total of 130 (in 2014) and 122 (in 2015) participating outlets were selected by simple random sampling across both domains and screened for inclusion. Eligible outlets were those with diagnostic services available on the day of survey, defined as offering an RDT service or reporting the availability of a functioning microscope and supplies of slides and stain. All 130 selected outlets were eligible in 2014 and 120 out of 122 were eligible in 2015. In 2014 data were collected by one research assistant over 3 days at each eligible outlet in Kwale and over 2 days in Kilifi/Mombasa; in 2015 data collection proceeded at each site for between 1 and 6 days (Kwale median: 6 days [IQR 5–6]; Kilifi/Mombasa median: 4 days [IQR 3–4]). At outlets with a high client load, multiple enumerators screened and interviewed clients.

#### Training and data collection

Prior to each round of data collection, research assistants and supervisors with experience in quantitative fieldwork involving pharmaceutical products were recruited and trained over 5 days by PS Kenya research staff. Training included a 1-day fieldwork practice session during which research assistants piloted all study procedures at a location outside the fieldwork area. Standard questionnaires were developed by a core team as part of a larger multi-country project. These were adapted to the Kenyan context, including translation to Swahili, and pilot tested in Kenya prior to the survey. Data collection used paper questionnaires in 2014 and KoboToolbox (Harvard Humanitarian Initiative, Cambridge, MA) in 2015. The client exit interview used three data collection instruments: an outlet screening tool, client exit questionnaire, and provider questionnaire.

On the first day of data collection at a new outlet, research assistants introduced the study to the outlet owner or most senior member of staff present and explained they were investigating “adult and child health care in this community”. Verbal consent of the outlet staff was sought before proceeding. The outlet screening questionnaire was administered to determine outlet eligibility and collect availability data on a broad range of essential medicines, diagnostic services and equipment, to conceal the malaria focus of the study. At eligible outlets, all clients leaving an outlet during opening hours were screened for inclusion. Eligible clients were adults seeking treatment for fever for themselves or on behalf of someone else. Respondents aged under 18, and cases where the patient was less than 2 months old, currently pregnant by self-report, or had been referred for serious illness were excluded. Verbal consent was obtained from eligible respondents before the interview, and interviews took place in a discreet location away from the main entrance to the outlet. The client exit interview covered client and patient demographics, prior malaria diagnosis and treatment sources, testing and treatment prescribed or received at the project outlet, counselling and advice received, client satisfaction, and household characteristics and asset ownership. Information on diagnostic testing for malaria was self-reported, while information on medicines prescribed or received during the consultation was captured from prescriptions or medicine packaging when available. Medicine types were coded on the questionnaire during the interview and cross-checked against recorded brand names during analysis. Median interview time in 2014 was 20 min [IQR 17–24] (data not available for 2015). At outlet closing time on the final day at each outlet, a short provider questionnaire was administered to either the dispensary staff member responsible for performing blood tests for clients (in a registered pharmacy) or the main provider in the dispensary if a health facility has a separate laboratory. The questionnaire covered knowledge and beliefs about diagnostic testing and treatment for malaria (results from this questionnaire are not presented here). During data collection, research assistants were visited and monitored regularly by team supervisors who also reviewed completed questionnaires to check for completeness, correct coding of questions and general logic. Data collected on paper questionnaires in 2014 were double entered in Microsoft Office Excel.

### Mystery client visits

#### Study design, sample size and sampling

Repeat cross-sectional mystery client visits to private health facilities and registered pharmacies were conducted in October 2014 and November–December 2015, following completion of the exit interview studies. The sampling frames for each round were those used for the exit interview study, described above. The study’s objective was to monitor the provision of anti-malarials to clients testing negative for malaria. Sample sizes were calculated to provide estimates of the proportion of clients presenting with self-reported recent febrile symptoms who test negative for malaria that did not receive any anti-malarial, by geographic domain, with a confidence level of 95% and a margin of error of 8 percentage points, assuming 30% of test-negative clients did not receive any anti-malarial. These calculations led to required samples of 126 mystery client visits in each domain, and 42 test-negative participants willing to undergo three finger pricks were recruited per domain for each study round. In line with the study objective, eligible client visits were defined as those where testing was available on the day of visit thus ensuring that the client could be tested for malaria. However, as availability of testing could not be confirmed prior to a mystery client visit, outlets were oversampled to ensure that sufficient outlets with testing services available were identified. In total, 155 (in 2014) and 113 (in 2015) participating outlets were selected by simple random sampling across both domains and visited by at least one mystery client. On average, participants visited two outlets over a 1-week period where they received a finger-prick for malaria testing (in both rounds). Written consent for an outlet to be included in the study was sought from the owner or senior staff member at the time of provider training or in advance of fieldwork (as per PSI’s policy on conducting mystery client studies).

#### Training and data collection

Participants were recruited from education, community and religious groups in the project areas. The study team strived to recruit participants with a range of backgrounds and ages, and a mix of genders. Participants had to be aged 18 years and above and have had no (self-reported) febrile illness in the 4 weeks prior to recruitment. Supervisors were recruited from PS Kenya’s pool of research staff. All participants and supervisors underwent a practical three-day training course, comprising multiple role-plays and observations of correct RDT procedure. RDTs were conducted on consenting participants and research staff so that best practice could be highlighted and actions during the testing procedure linked to follow-up questions on the questionnaire. Following training, a medically qualified study team member tested all participants by quality-assured RDT [*CareStart Malaria HRP2 (Pf)* manufactured by AccessBio (catalogue number G0141)] and confirmed their malaria test-negative status. All participants provided written consent to undergo blood testing prior to each mystery client visit. Standard post-visit questionnaires were developed, adapted and fielded as per the exit interview study.

On survey days each participant visited a pre-selected outlet and enacted the role of a patient who had suffered from fever the previous night. If the provider offered to perform a blood test the participant consented, was tested, and bought all medicines suggested by the provider (or obtained a prescription). If the provider did not offer a malaria blood test, the participant prompted for one in a natural manner, and following the test, proceeded to buy any medicines suggested by the provider. After exiting the outlet, the participant was met by a team supervisor who guided them through a short questionnaire covering the initial provider consultation, the process of getting tested, and information on any medicines received or prescribed. Medicine details were recorded as in the exit interview study. Participants were reimbursed the costs of consultation fees and charges for testing and medicines (though limited funds in 2015 meant that volunteers were not always able to purchase the medicines recommended by the providers).

#### Study outcome measures and analysis

The primary objective of this analysis was to describe private provider adherence to standard fever case management algorithms, and compare case management at participating private health facilities and registered pharmacies. First, exit interview data were used to determine the proportion of interviewed clients tested for malaria at active project outlets. As some interviewed clients were visiting the outlet on behalf of the patient (either with the patient in attendance or not) the proportion of eligible patients tested for malaria was estimated, with *eligible patient* defined as a patient who was present at the visit and reported not having been previously tested for malaria for this fever episode. Levels of testing were disaggregated into microscopy and RDT by client recall of the test type. Second, the proportion of patients who received a given type of medicine by test result was determined. Third, measures of provider counselling and advice were estimated based on client recall of the consultation. Mystery client data were then used to further examine possible deviant provider behaviour when faced with a negative blood test for malaria. First, the proportion of mystery clients who received the correct diagnosis and who additionally went on to not receive any anti-malarial was calculated. A *correct diagnosis* was defined as a client being told they were negative for malaria following testing. A broader range of quality of care measures was then assessed, covering client testing and aspects of test procedure as recalled by the mystery client.

For both studies, classification of medicines was based on local knowledge of brand names and was performed during analysis by a team member with experience working with anti-malarials in Kenya. Medicines were coded for analysis as artemisinin-based combination therapy (ACT), all other anti-malarials (all non-ACT), and antibiotics. Other medicine types were captured but are not reported in this analysis. Client socioeconomic status was captured through the client questionnaire using household asset questions derived from the Kenya 2011 Malaria Indicator Survey. Wealth quintiles were calculated based on the first component score from a principal components analysis conducted separately for each survey round based on data from clients at all outlets. Malaria diagnostic services and equipment, medicines and guidelines were considered *available* based on provider report or observation by the enumerator. Variables for each survey round for private health facilities and registered pharmacies were estimated separately. Cases missing data on any test received were excluded from the analysis of diagnostic test prevalence. Cases missing the information required to identify medicine types were excluded from analysis of medicine provision based on test result.

Point estimates used survey weights to account for the explicit domain stratification. For the exit interview survey, samples were additionally weighted to account for differences in the length of data collection by outlet. Standard errors and 95% confidence intervals were calculated accounting for clustering of clients at outlets and the domain specification. Outlet-level variables (such as the availability of RDTs on the day of interview) did not require an adjustment for clustering. Comparisons between private health facilities and registered pharmacies were made for each round of data collection independently, using the design-based F-test statistic. All data were reviewed and analysed using Stata v13 (StataCorp, College Station, TX).

### Ethics statement

Ethical approval for both studies was obtained from the AMREF Ethics and Scientific Review Committee in September 2014 (Ref: P131/2014) and February 2015 (Ref: P160/2015).

## Results

### Exit interview sample description

Table 1 presents the outlet and client study samples for private health facilities and registered pharmacies for the two exit interview rounds. In summary, 534 and 633 clients were interviewed at 130 and 120 eligible outlets in 2014 and 2015 respectively, with 455 and 541 patients present at the interview and not previously tested for malaria during the current fever episode. The project experienced relatively high outlet attrition, with 54% of enrolled outlets dropping out over 18 months, the majority of these being registered pharmacies. While similar numbers of active outlets were selected for the study in both years, 21 outlets left the project between survey rounds resulting in relatively fewer registered pharmacies in the 2015 sample compared with 2014. In 2015, the study team was unable to screen 6 outlets for inclusion (3 closed during the entire study period, 3 refusals). More eligible clients were recruited from private health facilities in both rounds [2014: 37% (417/1122) of clients screened in private health facilities were eligible, vs. 23% (122/542) at registered pharmacies; 2015: 26% (501/1915) of clients screened in private health facilities were eligible, vs. 15% (150/994) at registered pharmacies]. The median number of clients interviewed per eligible outlet was 4 at private health facilities in both rounds and at registered pharmacies in 2015, and 3 at registered pharmacies in 2014.

**Table 1:** Outlet and client sample description by survey round and outlet type

Description	2014			2015		
	Private health facility (N)	Reg’d pharmacy (N)	All outlets (N)	Private health facility (N)	Reg’d pharmacy (N)	All outlets (N)
Outlet sample						
Outlets ever enrolled in project	142	175	317	142	175	317
Outlets active in the project at the time of fieldwork^b^	106	61	167	–^a^	–^a^	146
Outlets selected for the study	86	44	130	–^a^	–^a^	128
Outlets screened	86	44	130	89	33	122
Outlets eligible^c^	86	44	130	89	31	120
Client sample						
Clients approached for inclusion	1133	551	1684	1970	1053	3023
Clients screened	1122	542	1664	1915	994	2909
Clients eligible	417	122	539	501	150	651
Clients interviewed^d^	413^e^	121	534	493	140	633
Patients < 5 years	100	24	124	173	35	208
Patients 5 years and older	311	97	408	320	105	425
Clients interviewed not previously tested for this febrile episode	374^f^	81	455	450	91	541
Patients < 5 years	88	12	100	158	20	178
Patients 5 years and older	285	69	354	292	71	363
Average number of clients interviewed per facility at which clients were interviewed (mean)	4.9	3.2	4.4	5.9	5.0	5.7
Average number of clients interviewed per facility at which clients were interviewed (median)	4 [2–6]	3 [1–5]	4 [2–6]	4 [2–8]	4 [3–8]	4 [2–8]

^a^Due to staff turnover, this information is not available

^b^Outlets were considered *active* if, at the time of data collection, they had placed at least one order for RDTs through the project

^c^Eligible outlets were those with diagnostic services available on the day of survey, defined as offering an RDT service or the reported availability of a functioning microscope and supplies of slides and stain

^d^Interviewed means that the client questionnaire is complete, or the interview was interrupted after questions about diagnostic testing had been answered

^e^2 cases at private health facilities are missing information on client age

^f^1 case at a private health facility is missing information on client age

### Exit interview outlet and patient characteristics

There were notable variations in outlet characteristics in both survey rounds (Table 2). Registered pharmacies (100%) were more likely than private health facilities (87.5%) to have RDTs available in 2014 (p = 0.01), though no difference was seen between outlet types in 2015 (96.6% of facilities vs. 100% of registered pharmacies, p = 0.297). By contrast, and as expected, private health facilities were more likely to have a functioning microscope available in both survey rounds (2014: 83.4% of facilities vs. 1.9% of registered pharmacies, p < 0.001; 2015: 77.5% of facilities vs. 6.4% of registered pharmacies, p < 0.001). No differences were seen between outlet types in either round in the availability of artemether–lumefantrine tablets, the first-line treatment for uncomplicated malaria in Kenya (2014: 97.7% of facilities vs. 100% of registered pharmacies, p = 0.306; 2015: 97.8% of private health facilities vs. 100% of registered pharmacies, p = 0.402). Private health facilities were consistently more likely to possess written guidelines on either the Integrated Management of Childhood Illness (IMCI) or on malaria diagnosis and treatment (2014: 96.7% of facilities vs. 79.2% of registered pharmacies, p = 0.001; 2015: 97.8% of facilities vs. 77.4% of registered pharmacies, p < 0.001).

**Table 2:** Patient and outlet characteristics by survey round and outlet type

Characteristics	2014			2015		
	Private health facility	Reg’d pharmacy	p-value	Private health facility	Reg’d pharmacy	p-value
	%	%		%	%	
Proportion of eligible outlets with	**N** **=** **86 outlets**	**N** **=** **44 outlets**		**N** **=** **89 outlets**	**N** **=** **31 outlets**	
Malaria RDT testing	87.5	100	0.012	96.6	100	0.297
Functioning microscopy	83.4	1.9	< 0.001	77.5	6.4	< 0.001
Thermometer	98.8	74.4	< 0.001	100	87.1	0.001
Child scale	72.7	9.0	< 0.001	74.2	3.2	< 0.001
AL tablets	97.7	100	0.306	97.8	100	0.402
Government guidelines for IMCI or diagnosis and treatment of malaria	96.7	79.2	0.001	97.8	77.4	< 0.001
Sharps container	100	92.8	0.015	100	100	–
Patient characteristics	**N** **=** **413**	**N** **=** **121**		**N** **=** **493**	**N** **=** **140**	
Patient present						
Yes	99.4	74.9	< 0.001	99.5	85.1	< 0.001
Patient gender^a^						
Male	43.8	55.7	0.015	49.1	57.1	0.074
Patient age (years)^b^						
0–4	24.9	20.4	0.293	36.6	25.4	0.080
≥ 5	75.1	79.6		63.4	74.6	
Patient/client education level^c^						
None	12.7	4.1	0.027	20.5	17.5	0.364
Primary	36.2	28.5		38.3	32.7	
Secondary	29.4	38.3		28.6	37.8	
University/Tertiary	21.7	29.1		12.7	12.0	
Patient/client socioeconomic status^d^						
Lowest	17.8	11.1	0.022	21.3	13.5	0.132
Second	19.6	10.1		20.6	13.3	
Middle	20.3	21.8		20.7	20.9	
Fourth	23.5	21.4		20.3	21.1	
Highest	18.9	35.6		17.2	31.3	
Previous treatment sought for this fever episode^e^						
Yes	18.2	31.8	0.005	20.0	40.2	< 0.001
Previous blood test during this fever episode^f^						
Yes	7.7	16.4	0.020	8.2	24.4	< 0.001
Symptoms on day of interview^g^						
Fever	82.4	80.6	0.708	85.5	75.6	0.161
Cough	32.3	28.1	0.490	36.2	31.6	0.413
Diarrhoea	9.8	10.7	0.809	14.1	9.0	0.272

^a^1 case at a private health facility (2014) and 1 case at a registered pharmacy (2015) missing information on client sex

^b^2 cases at private health facilities (2014) are missing information on client age

^c^1 case at a private health facility (2014) and 1 case at a private health facility (2015) are missing information on client education level

^d^10 cases at private health facilities and 4 cases at registered pharmacies (2014); 9 cases at private health facilities and 4 cases at registered pharmacies (2015) are missing information on socioeconomic status

^e^1 case at a private health facility (2015) missing information on previous treatment sought

^f^1 case at a private health facility (2015) missing information on previous blood test

^g^1 case at a private health facility (2015) missing information on fever, coughing and diarrhoea symptoms

Patient characteristics also varied by outlet type in both survey rounds. Patients at private health facilities were more likely to be female and to be present at the client interview than those at registered pharmacies. Patients were present at over 99% of interviews at private health facilities in 2014 and 2015, compared with 74.9% of interviews at registered pharmacies in 2014 (p < 0.001) and 85.1% of interviews in 2015 (p < 0.001). Registered pharmacy patients (or the clients attending on their behalf) were more likely to belong to higher wealth categories than those surveyed at private health facilities in 2014, though no difference was observed in 2015 (2014: p = 0.022; 2015: p = 0.132). As anticipated, registered pharmacy patients were more likely than those at private health facilities to have sought care from another source during this fever episode and more likely to have already received a blood test for malaria during this fever episode (2014: 16.4% at registered pharmacies vs. 7.7% at private health facilities, p = 0.02; 2015: 24.4% at registered pharmacies vs. 8.2% at private health facilities, p < 0.001). No significant differences between outlet types were noted in the proportion of patients reporting fever, cough or diarrhoea at the time of interview in either round.

### Level of patient diagnostic testing

According to national guidelines, all patients presenting with fever or with a history of fever should receive a blood test for malaria. Among all visits in 2014, 84.1% of private health facility clients reported receiving a blood test for malaria, compared with 39.7% of registered pharmacy clients (p < 0.001) (Table 3); similar levels were reported in 2015. Eligible patients were defined as patient who was present at the visit and reported not having been previously tested for malaria for this fever episode. Among eligible patients at registered pharmacies the reported level of testing was 60.8% in 2014 and 56.3% in 2015, significantly lower than testing among eligible patients at private health facilities (85.6% in 2014, p < 0.001; 85.3% in 2015, p < 0.001). Eligible patients were more likely to report being tested by RDT in registered pharmacies than in private health facilities in 2014 (52.1% vs. 30.4%, p = 0.013), though no difference was seen in 2015 (56.3% vs. 52.6%, p = 0.724). Testing by microscopy was more common in private health facilities than registered pharmacies in both rounds (2014: 35.6% at private health facilities vs. 4.7% at registered pharmacies, p = 0.006; 2015: 27.3% at private health facilities vs. 0% at registered pharmacies, p = 0.014). In 2014, 17.8% of eligible private health facility patients were unable to recall the type of test they received. On average 48% of patients that received an RDT were reportedly told they were positive for malaria across all outlets and rounds, with no significant difference observed between outlet types in reported RDT test result in either round. The median reported cost for an RDT was 100 Kenyan Shillings (about USD 0.88 in 2014 and USD 0.99 in 2015) in both outlet types in both survey rounds. Among 178 eligible patients across both rounds who did not receive a test, the most frequently stated reasons at both outlet types were related to presumptive diagnosis—“*I/the doctor know(s) I don’t have malaria”* (14.6%, n = 26); “*I/the doctor know(s) it is malaria”* (12.4%, n = 22)—followed by “*a test wasn’t offered”* (26.4%, n = 47), and then *“cost”* (16.3%, n = 29).

**Table 3:** Case management of patients by survey round and outlet type

Description	2014			2015		
	Private health facility	Registered pharmacy	p-value	Private health facility	Registered pharmacy	p-value
	%	%		%	%	
Malaria blood testing^a^	**N** **=** **413**	**N** **=** **121**		**N** **=** **493**	**N** **=** **139**	
Patient tested for malaria	84.1	39.7	< 0.001	83.2	40.0	< 0.001
Eligible patient^b^	**N** **=** **374**	**N** **=** **81**		**N** **=** **450**	**N** **=** **91**	
Tested for malaria	85.6	60.8	0.001	85.3	56.3	< 0.001
Tested by RDT	30.4	52.1	0.013	52.6	56.3	0.724
Tested by microscopy	35.6	4.7	0.006	27.3	0.0	0.014
Tested, test type unknown	17.8	4.0	0.016	5.1	0.0	0.189
Reported malaria test result						
Proportion of patients tested by microscopy that reported	**N** **=** **138**	**N** **=** **6**		**N** **=** **141**	**N** **=** **0**	
Result was positive for malaria	51.6	50.0	0.810	63.4	–	–
Proportion of patients tested by RDT that reported	**N** **=** **134**	**N** **=** **41**		**N** **=** **250**	**N** **=** **58**	
Result was positive for malaria	52.3	47.2	0.338	45.1	52.8	0.712
Medicines bought/prescribed^c^						
Proportion of test positive patients that received any^d^	**N** **=** **191**	**N** **=** **25**		**N** **=** **203**	**N** **=** **35**	
Medicine	97.1	86.8	0.074	98.0	97.1	0.750
Any anti-malarial	84.6	86.8	0.831	91.6	92.6	0.843
ACT	75.5	86.8	0.343	84.1	85.6	0.841
Other anti-malarial	12.0	0.0	0.249	13.4	6.9	0.401
Antibiotic	40.4	21.1	0.090	47.0	14.2	0.004
Anti-malarial and antibiotic	30.9	21.1	0.358	42.3	11.8	0.001
Proportion of test negative patients that received any	**N** **=** **151**	**N** **=** **23**		**N** **=** **206**	**N** **=** **23**	
Medicine	85.3	69.6	0.124	92.4	88.6	0.471
Any anti-malarial	6.7	13.9	0.419	3.9	0.0	0.671
ACT	5.0	13.9	0.255	2.2	0.0	0.667
Other anti-malarial	2.0	0.0	0.521	2.5	0.0	0.711
Antibiotic	63.2	33.5	0.058	69.6	40.3	0.027
Anti-malarial and antibiotic	4.2	11.2	0.387	1.8	0.0	0.662
Proportion of untested eligible patients that received any	**N** **=** **48**	**N** **=** **31**		**N** **=** **60**	**N** **=** **38**	
Medicine	92.7	89.8	0.673	95.0	89.5	0.335
Any anti-malarial	8.4	40.8	0.001	19.8	22.2	0.807
ACT	8.4	34.7	0.005	4.9	13.5	0.143
Other anti-malarial	0.0	6.1	0.051	11.6	10.6	0.884
Antibiotic	68.7	24.6	0.001	50.5	31.8	0.069
Anti-malarial and antibiotic	1.2	4.1	0.323	5.3	5.5	0.974
Provider counselling and advice^e^						
Proportion of clients that were told to	**N** **=** **413**	**N** **=** **121**		**N** **=** **493**	**N** **=** **140**	
Come back immediately if the condition gets worse	14.5	14.8	0.944	33.8	17.6	0.026
Come back in 2 days if there is no improvement	6.9	5.2	0.619	10.6	11.3	0.900

^a^1 case at a registered pharmacy missing information

^b^An eligible patient is a patient who was present and had not been previously tested for malaria for the fever episode prior to the visit

^c^Cases missing information: in 2014, 3 at private health facilities and 3 at registered pharmacies; in 2015, 8 cases at private health facilities and 1 at a registered pharmacy

^d^Patients could receive or be prescribed more than one medicine and totals do not sum to 100%

^e^Cases missing information: in 2014, 4 at private health facilities and 4 at registered pharmacies; in 2015, 6 at private health facilities and 3 at registered pharmacies

### Treatments received by patients by test status

At least 75% of all test-positive patients in both rounds bought or were prescribed ACT, with no significant difference between outlet types in either round. More than one in ten test-positive health facility patients received another type of anti-malarial in 2014 (12.0%) and 2015 (13.4%). Prescriptions and sales of antibiotics were significantly more common among test-positive health facility patients compared with test-positive registered pharmacy patients in 2015, though the difference was only borderline significant in 2014 (2014: 40.4% at private health facilities vs. 21.1% at registered pharmacies, p = 0.09; 2015: 47.0% at private health facilities vs. 14.2% at registered pharmacies, p = 0.004). Both rounds also saw a substantial proportion of test-positive patients receive both an anti-malarial and an antibiotic, with this practice seemingly more common in private health facilities than registered pharmacies (2014: 30.9% at private health facilities vs. 21.1% at registered pharmacies, p = 0.387; 2015: 42.3% at private health facilities vs. 11.8% at registered pharmacies, p = 0.001).

The provision of anti-malarials to test-negative patients was uncommon. The highest proportion of test-negative patients receiving any anti-malarial was seen in registered pharmacies in 2014 at 13.9% (3/23). Test-negative private health facility patients were at least 1.7 times as likely as registered pharmacy patients to receive an antibiotic (2014: 63.2% at private health facilities vs. 33.5% at registered pharmacies, p = 0.058; 2015: 69.6% at private health facilities vs. 40.3% at registered pharmacies, p = 0.027). Combined receipt of an anti-malarial and antibiotic was generally low due to the low level of anti-malarial receipt among test-negative patients.

A small number of eligible patients reported not being tested, and anti-malarial treatments received by these patients varied by outlet type and year. While a significantly higher proportion of untested registered pharmacy patients than private health facility patients received an anti-malarial in 2014 (40.8% vs. 8.4%, p = 0.001), no difference was seen in 2015 (22.2% vs. 19.8%, p = 0.807). The receipt of an antibiotic was significantly higher among untested private health facility clients than untested registered pharmacy clients in 2014 (68.7 vs. 24.6%, p = 0.001) with some suggestion of a persistent difference observed in 2015 (50.5% vs. 31.8%, p = 0.069).

### Patient counselling and advice

Clients were asked to spontaneously recall any messages or advice they had received from the provider(s) during their visit. Reports of advice received were mixed but overall infrequent. In 2014, less than 15% of clients at either outlet type recalled being advised to return immediately if their condition got worse. A similar level was seen among registered pharmacy patients in 2015 (17.6%) while among private health facility patients it was 33.8% (p = 0.026). Reported recall of advice to come back in 2 days if there is no improvement was universally under 12% and not significantly different across outlet types in either round.

### Mystery client sample description

Table 4 presents outlet study samples for the two rounds of mystery client visits. In summary, 83 mystery clients made 260 visits to 155 outlets in 2014, and 84 mystery clients made 262 visits to 113 outlets in 2015. Diagnostic testing services were unavailable for 46/260 visits in 2014 and 12/262 visits in 2015, resulting in 214 and 250 eligible client visits made in 2014 and 2015, respectively.

**Table 4:** Outlet sample and client visit description by survey round and outlet type

Description	2014			2015		
	Private health facility (N)	Reg’d pharmacy (N)	All outlets (N)	Private health facility (N)	Reg’d pharmacy (N)	All outlets (N)
Outlet sample						
Outlets ever enrolled in project	142	175	317	142	175	317
Outlets active in the project at the time of fieldwork^b^	106	61	167	–^a^	–^a^	146
Outlets approached by mystery clients	97	58	155	73	40	113
Outlets at which eligible client visits were made^d^	91	38	129	72	39	111
Client visits						
Number of mystery client participants	–	–	83	–	–	84
Client approaches	167	93	260	168	94	262
Visits during which testing was not available^c^	11	35	46	4	8	12
Eligible client visits^d^	156	58	214	164	86	250

^a^Due to staff turnover, this information is not available

^b^Outlets were considered *active* if, at the time of data collection, they had placed at least one order for RDTs through the project

^c^Testing was considered not available if in outlets that typically test by RDT there were no RDTs in stock on the day of interview and/or in outlets that typically test by microscopy there was no microscopy testing available due to a lack of staff or auxiliary materials

^d^Eligible client visits are visits to outlets with testing confirmed as available ex-post

### Mystery client visit outcomes

**Fig. 1:**
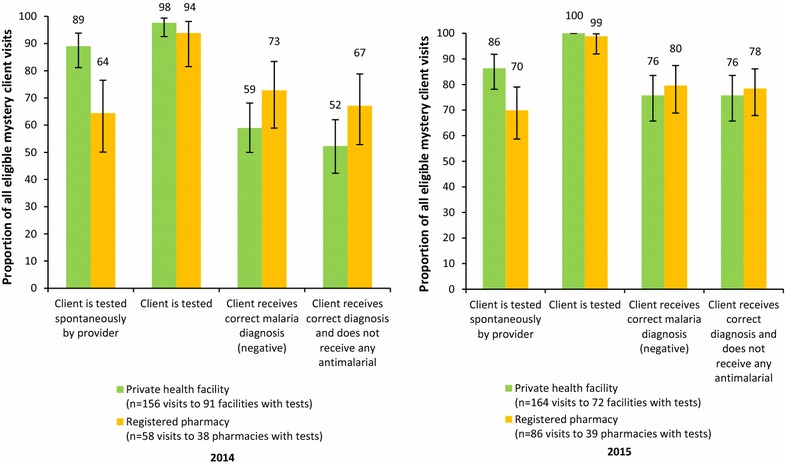
Mystery client visit outcomes by survey round and outlet type. *Tested spontaneously* means that the client was tested without having to prompt or request a test be conducted. Missing data: In 2014, 3 cases at private health facilities and 1 case at a registered pharmacy missing test results excluded from analysis; in 2015, 4 cases at private health facilities and 2 cases at registered pharmacies missing test results excluded from analysis

Mystery client visit outcomes for key steps on the fever case management algorithm are shown in Fig. 1. In both rounds, mystery clients were more likely to be tested spontaneously (i.e. without prompting) at private health facilities compared with registered pharmacies (2014: 89.0% at private health facilities vs. 64.4% at registered pharmacies, p < 0.001; 2015: 86.3% at private health facilities vs. 69.9% at registered pharmacies, p = 0.008). Mystery clients were trained to request a test if one was not offered by the provider: in total, clients were tested during at least 94% of visits, with no difference between outlet types in either round. In both rounds, relatively more mystery clients at registered pharmacies received the correct negative diagnosis than those at private health facilities, though outlet differences were not significant in either round. Over the study rounds, between 20% and 41% of visits resulted in a client being told they were positive for malaria by the provider. The composite endpoint of the mystery client visits was the proportion of visits at which the client received a correct negative diagnosis and did not receive any anti-malarials. In 2014, this was achieved in 67.1% visits to registered pharmacies and 52.3% of health facility visits (p = 0.089). In 2015, the level had increased in both outlet types and the gap between types had narrowed, with 78.4% of registered pharmacy visits and 75.7% of health facility visits receiving a correct negative diagnosis and no anti-malarials (p = 0.68).

### Provider competence for malaria blood testing of mystery clients

Mystery client volunteers were trained to observe and recall provider actions at key steps during the blood testing process, and adherence to quality of care standards varied across outlet types in both survey rounds (Table 5). Mystery clients reported that providers cleaned the mystery client patient’s finger with an alcohol swab and (independently) told the client the test result in over 95% of visits with testing, with no difference in the prevalence of these behaviours between outlet types in either round. Test areas were perceived as clean in at least eight out of ten visits. The provider immediately disposed of the lancet in a sharps bin on 75–82% of occasions, with no significant difference between outlet types in either round. The use of a separate area for conducting the test was significantly less common in registered pharmacies in both rounds, (2014: 72.9% at private health facilities vs. 40.7% at registered pharmacies, p < 0.001; 2015: 76.9% at private health facilities vs. 39.8% at registered pharmacies, p < 0.001). Registered pharmacy staff were significantly more likely to wear gloves when performing the test than were staff at private health facilities, with gloves worn for less than four in ten private health facility tests for both rounds (2014: 23.9% at private health facilities vs. 63.3% at registered pharmacies, p < 0.001; 2015: 37.9% at private health facilities vs. 66.8% at registered pharmacies, p = 0.011). When observing RDT performance specifically, providers rarely placed the blood or buffer in the wrong wells on the RDT. The poorest performance was recorded in registered pharmacies in 2014 when blood was placed incorrectly in 8.3% (4/48) of RDTs and buffer was placed incorrectly in 6.3% (3/48) of RDTs; however, there was no significant difference between outlet types in this behaviour in either round. RDT results should be read 15–20 min after the buffer has been added to the cassette. In 2014, providers read the test result before 15 min had passed for 50.4% of tests in private health facilities and in 63.9% of tests in registered pharmacies (p = 0.217). These levels decreased in both outlet types in 2015 but most RDTs were still read too soon in registered pharmacies (57.8%), when compared with the level in private health facilities (37.3%, p = 0.031).

**Table 5:** Provider quality of care related to diagnostic testing by survey round and outlet type

Description	2014			2015		
	Private health facility	Reg’d pharmacy	p-value	Private health facility	Reg’d pharmacy	p-value
	%	%		%	%	
Proportion of visits including a test at which the provider	**N** **=** **152**	**N** **=** **54**		**N** **=** **164**	**N** **=** **85**	
Explained how the test would be conducted	25.3	33.3	0.338	14.7	20.7	0.208
Performed the test in a separate area (away from other clients)	72.9	40.7	< 0.001	76.9	39.8	< 0.001
Performed the test in a clean area	80.4	90.9	0.097	90.6	95.4	0.184
Wore gloves while performing test	23.9	63.3	< 0.001	37.9	66.8	0.011
Cleaned the mystery client’s finger with an alcohol swab	96.8	100	0.182	95.9	95.0	0.807
Immediately disposed of the lancet in a sharps bin	76.7	75.3	0.859	78.9	81.5	0.662
Told the client the test result	97.8	98.0	0.926	97.4	97.8	0.877
Proportion of visits including an RDT at which the provider	**N** **=** **63**	**N** **=** **48**		**N** **=** **90**	**N** **=** **82**	
Placed the blood in the correct hole in the RDT^a^	96.8	91.0	0.201	97.9	97.7	0.927
Placed the buffer in the correct hole in the RDT^b^	96.3	93.1	0.513	100	97.7	0.154
Waited less than 15 min before reading the result	50.4	63.9	0.217	37.3	57.8	0.031

^a^Cases that did not observe where blood was placed: in 2014, 1 at a health facility and 2 at registered pharmacies; in 2015, 2 at private health facilities and 2 at registered pharmacies

^b^Cases that did not observe where buffer was placed: in 2014, 3 at private health facilities and 3 at registered pharmacies; in 2015, 4 at private health facilities and 3 at registered pharmacies

## Discussion

Registered pharmacies in Kenya are not legally permitted to perform RDTs and an evidence-base is required to inform active policy discussions in this area. This paper aims to contribute to this evidence-base. Results from this analysis show that for many quality-of-care indicators, registered pharmacy providers’ RDT performance and compliance to test results is comparable to that of staff in private health facilities, where RDT services are already permitted alongside microscopy. These findings come from an implementation setting where project interventions (including training, supportive supervision, feedback, and the supply of RDTs and accessories) were the same for outlets and providers in both channels. The expansion of RDT services to registered pharmacies in Kenya holds the promise of increasing access to high-quality malaria diagnosis, especially considering the pharmacies’ known role as a treatment source for childhood fever and as a source of anti-malarials.

Exit interview data suggest that RDT use in registered pharmacies was of a similar level to that in private health facilities, with 50% of eligible febrile patients tested by RDT. Both the exit interview and mystery client studies indicate that febrile patients at private health facilities were more likely to receive any diagnostic test for malaria. This is unsurprising given the additional availability of microscopy in the private health facility setting, together with the laboratory technicians who are permitted to perform these tests. Though the data sources are not strictly comparable, it appears that RDT use at participating registered pharmacies increased rapidly and was sustained over the life of the project when compared to the 4% testing level reported from the unpublished 2013 household survey and the low availability of RDTs recorded in pharmacies during the 2013 mapping exercise.

The use of RDTs in registered pharmacies in this study falls in the middle of the broad range of results identified in a recent systematic review of RDT introduction in the private retail sector (5 of 11 studies reported uptake below 50% with the remaining 6 studies reporting uptake of 50% and above) [8]. The private health facility results reported here compare favourably to results from public health facilities in Kenya, where 76% of febrile patients were tested at facilities with both microscopy and RDTs available [5]. Unlike experiences in other RDT intervention studies [8, 9], RDT cost was not a commonly stated barrier to RDT use in either channel. Mystery client results for both years also support the idea that, when prompted for a test by the client and when testing is available, registered pharmacy staff are as likely to conduct a test as staff in private health facilities, and that almost all client requests lead to a test being conducted.

In this setting, registered pharmacy providers were just as likely to provide appropriate anti-malarial treatment by test result as were providers at private health facilities. The proportion of test-positive registered pharmacy patients receiving ACT (86%) was similar to levels reported from other studies in sub-Saharan Africa: Visser et al. found test-positive compliance for medicine retailers was over 85% in 6 out of 11 studies [8]. The proportion of test-negative registered pharmacy patients who did not receive any anti-malarial ranged from 86 to 100%, also in line with the findings of Visser et al. that compliance among medicine retailers was over 80% in 8 out of 11 studies [8]. Study results from private health facilities were slightly lower than the published literature: a recent meta-analysis of clinician performance found a test-positive compliance above 90% for 9 out of 10 studies, and a test-negative compliance of 75% (pooled proportion) [10]. However, within Kenya, the private health facility results compare favourably with recent results from public facilities [5].

A novel element of this study highlights concern around the diagnosis providers are reporting. Among known-negative mystery clients, 20–41% were told they were positive for malaria, with no difference between retail outlet types. Future research should seek to establish to what extent this gap can be attributed to sub-optimal skills in conducting and interpreting diagnostic tests, to the time taken for providers to trust RDT results, to providers’ needs to manage patient expectations, or to misaligned business incentives and overt misrepresentation of the test result. This analysis and unpublished project monitoring data suggest that, overall, providers quickly showed competence in performing RDTs during routine supervision visits, and that a lack of skills may not be the main driver of this novel finding.

Exit interview analysis identified a relatively high level of antibiotic prescription for all patients irrespective of their test result, and strong evidence that this practice was more common at private health facilities than at registered pharmacies. Across both rounds, antibiotic prescription for test-negative registered pharmacy patients ranged from 14 to 34% compared with 40–69% for private health facility patients. This might be expected given the availability of qualified staff trained to perform differential diagnosis in health facilities, however, these levels of antibiotic prescription are far higher than fever aetiology studies in the region would suggest as required for the background level of bacteraemia [11–14]. The results from registered pharmacies are broadly in line with those from Visser et al., who identified antibiotic prescription for RDT-negative cases above 20% in 3 out of 7 studies. The private health facility results are at the lower end of those reported from public facilities in the region [15, 16]. Recent evidence from a broad range of epidemiological and healthcare settings show higher use of antibiotics for malaria test-negative patients relative to test-positive patients [17], a finding generally supported by these results. Antibiotic overuse (in both test-negative and test-positive cases) needs to be addressed to reduce pressure on the development of antibiotic resistance [17, 18].

Comparable levels of adherence to key steps in the RDT procedure by registered pharmacy and private health facility providers were observed through the mystery client visits. However, critical steps related to blood safety and hazardous waste management were sub-optimal in both channels, and registered pharmacy providers were somewhat more likely to read results before 15 min had elapsed. These challenges have been seen in other studies of medicine retailers [8], public health workers in Uganda [19] and among community health workers (CHWs) in Zambia [20], though in a longitudinal CHW study in Zambia, performance started high and improved over time [21]. More effort needs to be made in ensuring all providers wait the required time before reading RDT results. In the pharmacy setting, where clients are used to a short interaction with the provider, patient pressure and expectations may cause pharmacists to read tests early.

While these findings suggest providers do comply with national treatment recommendations based on the reported test result, these studies identified cases of irrational use of anti-malarials following provider training and routine supportive supervision. Previous studies of RDT implementation among medicine retailers and health workers have highlighted a variety of reasons why RDT uptake and provider compliance may be suboptimal, over and above questions of commodity supply and price. These include the providers’ perceived need to satisfy patient expectations, provider confidence in the test result, health worker preference to diagnose based on clinical symptoms, providers’ concerns over patients’ conditions worsening when anti-malarials are withheld, and the available level of motivation and supportive supervision available to the provider [10, 22–29]. Separately, past malaria-focused interventions targeting medicine sellers have been successful when they included a situation analysis of the legal and market environment, sought buy-in from participants and government, and included ongoing supervision [26]. Uptake of RDTs and adherence to results is likely associated with, among other things, longer provider trainings and frequent supervision visits [8], and can be supported by an approach that seeks to better understand the priorities and capacities of providers [29]. These elements of success were all critical to the intervention design described here. Activities included an assessment of the existing private sector RDT market, partnership and negotiation with government agencies and professional bodies, and trialled novel cost-effective approaches to supervision planning and provider feedback. However, due to the nature of the implementation and evaluation approach, it is not possible to draw further conclusions on the relative importance of these individual components.

Future research, in this and other country contexts, should seek to explore the most effective mix of interventions to support private sector RDT implementation, and estimate the cost effectiveness of different approaches to inform ongoing and future discussion on sustainability. In addition, given the high levels of antibiotic prescription seen in this study, further research is needed to determine whether RDTs supported by IMCI training and supportive supervision can support better targeting of antibiotics for treatment for pneumonia and other major bacterial infections. In the absence of a point-of-care test for malaria, bacterial and viral illnesses [18], it is critical that the drivers of effectiveness of differential diagnosis following the introduction of RDTs for malaria are better understood.

### Limitations

These studies are not without their limitations. First, exit interview data on the test type and test result were based on client recall, and no observation or patient re-testing was performed. Results may be subject to recall bias, and the study recorded a relatively high level of *don’t know* responses (19%) for test type in private health facilities in 2014. However, across a range of malaria test-related outcomes, exit interviews have shown a good level of sensitivity and specificity in other settings [30]. Second, exit interview data collection occurred over several days at each outlet, while the availability of malaria diagnostic tests was only recorded on the first day (as part of screening), it is therefore possible that testing services were not always available during the data collection period. Third, although providers were blinded to the true focus of the study, it is possible that the continued presence of enumerators could have influenced provider practices during data collection. A recent analysis of exit interview results from public health facilities in Tanzania concluded there was some suggestion of modest improvements in provider behaviour during fieldwork periods [31]. Hypothesising that providers may correct their ‘usual’ level of compliance to test-negative cases, mystery client visits were used to address the possible Hawthorne effect.

Another limitation is the relatively small sample size achieved among some sub-groups in the exit interview study and the small sample deployed for the mystery client study. The mystery client study was restricted by the logistical difficulties inherent in attempting to recruit many volunteers willing to undergo multiple finger pricks. For the exit interviews, client load and overall samples were lower than anticipated in both survey rounds (despite increasing the number of fieldwork days in 2015), and limited by programme logistics. The exit interview study failed to meet the domain-specific sample size requirements in both rounds. To address these issues, this analysis was conducted independently for each exit interview and mystery client survey round and conclusions have been drawn based on the consistency of findings across study types and rounds. Only limited funds were made available to volunteers in the 2015 mystery client study and volunteers were not always able to purchase the medicines recommended by the providers. For this reason, mystery client results presented here are restricted to reported test result and provider competence in performing the diagnostic test.

Finally, caution must be taken before interpreting these results as representative of all private sector providers, due to the limited external validity of these studies. By restricting study eligibility to outlets active in the project, the sample is self-selecting based on willingness to engage in testing. Provider behaviour at these outlets may differ to behaviours at private outlets in general. These studies were designed to assess whether and how RDTs are used by providers *when available*. Additional data sources, such as facility and outlet surveys [7], remain important to provide contextual information on diagnostic availability. These results can be interpreted as upper limits for outcomes following training and routine supportive supervision to engaged providers in this setting. Further research is required on effective methods to identify providers likely to engage in such a project and to motivate private providers, particularly in pharmacies, to adopt RDTs when permitted.

## Conclusion

Taken together, these results from two independent rounds of observational studies suggest that non-laboratory private sector staff can use malaria RDTs in a real-world setting in Kenya in line with malaria control guidelines. Further, malaria testing and treatment outcomes from these non-laboratory staff were comparable to those at private health facilities in the same project, and similar to published results from the public sector in Kenya. These results can be used to advance the policy discussion in Kenya and in other settings on the role for registered pharmacies in providing universal access to malaria diagnostic testing.

## Abbreviations

AMFm: Affordable Medicines Facility-malaria
AMREF: African Medical Research Foundation
ACT: artemisinin-based combination therapy
AL: artemether–lumefantrine
BCC: behaviour change communication
CHW: community health worker
DFID: the UK Department for International Development
FIND: Foundation for Innovative New Diagnostics
IPC: Institute Pasteur of Cambodia
IMCI: Integrated Management of Childhood Illness
JHSPH: Johns Hopkins Bloomberg School of Public Health
KMLTTB: Kenya Medical Laboratory Technicians and Technologists Board
MOH: Ministry of Health
NMCP: National Malaria Control Programme
PPB: Pharmacy and Poisons Board
PS Kenya: Population Services Kenya
QAACT: quality-assured artemisinin-based combination therapy
RDT: rapid diagnostic test
RITM: Research Institute for Tropical Medicine
SOP: Standard Operational Procedures
USD: United States of America dollars
WHO: World Health Organization
